# Validity of a Novel Digitally Enhanced Skills Training Station for Freehand Distal Interlocking

**DOI:** 10.3390/medicina58060773

**Published:** 2022-06-07

**Authors:** Torsten Pastor, Tatjana Pastor, Philipp Kastner, Firas Souleiman, Matthias Knobe, Boyko Gueorguiev, Markus Windolf, Jan Buschbaum

**Affiliations:** 1 AO Research Institute Davos, 7270 Davos, Switzerland; philipp.kastner@aofoundation.org (P.K.); firas.souleiman@medizin.uni-leipzig.de (F.S.); boyko.gueorguiev@aofoundation.org (B.G.); markus.windolf@aofoundation.org (M.W.); jan.buschbaum@aofoundation.org (J.B.); 2 Department of Orthopedic and Trauma Surgery, Lucerne Cantonal Hospital, 6000 Lucerne, Switzerland; matthias.knobe@uzh.ch; 3 Department of Hand Surgery, Bern University Hospital, University of Bern, 8008 Bern, Switzerland; tatjana.pastor@insel.ch; 4 Department for Orthopaedics and Traumatology, Johannes Kepler University, 4021 Linz, Austria; 5 Department of Orthopaedics, Trauma and Plastic Surgery, University Hospital Leipzig, 04103 Leipzig, Germany; 6 Medical Faculty, University of Zurich, 8091 Zurich, Switzerland; 7 Medical Faculty, RWTH Aachen University Hospital, 52074 Aachen, Germany

**Keywords:** education, simulation, distal interlocking, intramedullary nailing, training

## Abstract

*Background and Objectives:* Freehand distal interlocking of intramedullary nails is technically demanding and prone to handling issues. It requires precise placement of a screw through the nail under fluoroscopy guidance and can result in a time consuming and radiation expensive procedure. Dedicated training could help overcome these problems. The aim of this study was to assess construct and face validity of new Digitally Enhanced Hands-On Surgical Training (DEHST) concept and device for training of distal interlocking of intramedullary nails. *Materials and Methods:* Twenty-nine novices and twenty-four expert surgeons performed interlocking on a DEHST device. Construct validity was evaluated by comparing captured performance metrics—number of X-rays, nail hole roundness, drill tip position and drill hole accuracy—between experts and novices. Face validity was evaluated with a questionnaire concerning training potential and quality of simulated reality using a 7-point Likert scale. *Results:* Face validity: mean realism of the training device was rated 6.3 (range 4–7). Training potential and need for distal interlocking training were both rated with a mean of 6.5 (range 5–7), with no significant differences between experts and novices, *p* ≥ 0.234. All participants (100%) stated that the device is useful for procedural training of distal nail interlocking, 96% wanted to have it at their institution and 98% would recommend it to colleagues. Construct validity: total number of X-rays was significantly higher for novices (20.9 ± 6.4 versus 15.5 ± 5.3, *p* = 0.003). Success rate (ratio of hit and miss attempts) was significantly higher for experts (novices hit: *n* = 15; 55.6%; experts hit: *n* = 19; 83%, *p* = 0.040). *Conclusion:* The evaluated training device for distal interlocking of intramedullary nails yielded high scores in terms of training capability and realism. Furthermore, construct validity was proven by reliably discriminating between experts and novices. Participants indicate high further training potential as the device may be easily adapted to other surgical tasks.

## 1. Introduction

Freehand distal interlocking of intramedullary nails is technically demanding and prone to handling issues. Young orthopaedic trauma residents usually acquire the skills necessary for this surgical step by a combination of observation, reading, and supervised guided operative experience [1]. However, increased efforts on administrative tasks and work-hour restrictions reduce the amount of time young surgeons can spend apprenticing with expert surgeons in the operating room [2,3,4]. Furthermore, the steadily growing workload of medical doctors [5,6] may have an impact on the time spent for teaching operations as they are time consuming and consequently more expensive. Therefore, medical teaching shifts towards hands-on exercises as new technologies allow for a more realistic training scenarios without endangering patients. Distal nail interlocking is a good example of this current problem as it is the most difficult part performed at the end of the surgery and, if not correctly conducted, may have a catastrophic impact on the patient’s outcome [7]. This particular surgical task requires up to a quarter of the operation time and one third of the overall radiation exposure [8], thus affecting not only the patient but also the whole operation team. Consequently, numerous techniques, aiming devices and navigation systems have been suggested to aid distal targeting, aiming to overcome some of the associated problems [9,10,11,12,13,14,15,16,17,18,19,20]. However, although radiation-independent devices exist, the freehand technique remains the predominant method used by orthopaedic trauma surgeons [9]. Furthermore, surgeons must still master the freehand technique to overcome technical errors with the targeting devices or navigation systems. Recently, a new Digitally Enhanced Hands-On Surgical Training (DEHST) concept was developed at the AO Research Institute Davos [21]. The pilot module allows training of distal nail interlocking by applying the freehand technique under simulated fluoroscopy guidance. The DEHST training concept features practical training with haptic feedback and performance evaluation over the learning process. So far, the effectiveness of this training technology has not been demonstrated. Furthermore, DEHST must reliably discriminate between experts and novices as the first steps of the validation cascade establish face and construct validity. Therefore, the aim of this study was to assess construct and face validity of the new DEHST concept and device for training of distal interlocking of intramedullary nails.

## 2. Materials and Methods

### 2.1. Digitally Enhanced Hands-On Surgical Training (DEHST)

DEHST is a novel modular, cost-effective and transportable system for surgical skills training and assessment. It augments haptic stations for hands-on training with digital technologies to enhance training scope and improve user experience. A proprietary optical tracking technology is utilized to allow position and orientation tracking in 6 degrees of freedom from a single planar image projection [22]. This tracking technology is adapted for use with a conventional video camera [23], enabling tracking of the specific movements during surgical tasks and providing comprehensive performance analysis in low-cost fashion. The modular nature of the system may facilitate building of a product line of modules targeting various surgical skills in traumatology and orthopaedics in the future.

**Figure 1:**
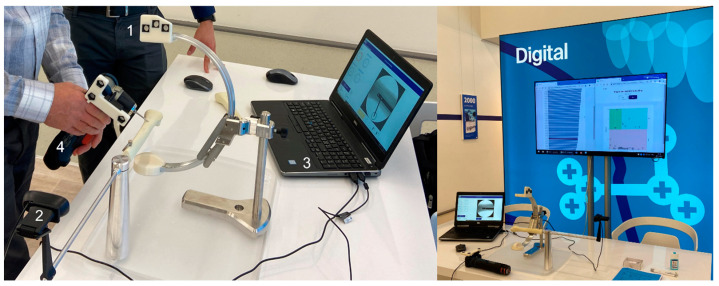
Digitally Enhanced Hands-On Surgical Training (DEHST) module for distal nail interlocking. 1: Model of a C-arm with reference markers for optical tracking; 2: Conventional optical camera; 3: Computer with imaging engine for simulation of X-ray images; 4: Angulated power drill with reference markers. Right: Training station at AO Davos Courses 2021 with score board presenting participants’ performance in relation to the tested peer group.

**Figure 2:**
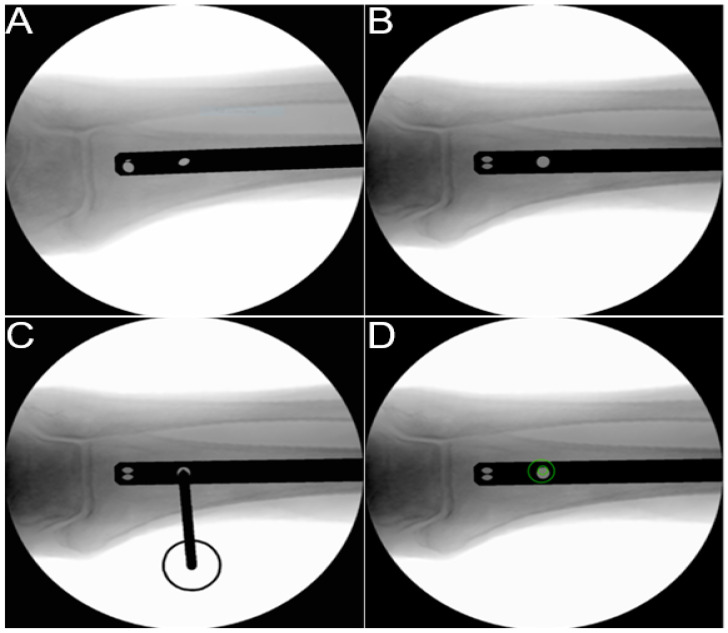
Sequence of simulated X-ray images of distal nail interlocking. (**A**) Arbitrary starting position; (**B**) “Perfect circle” hole projection after aligning the C-arm; (**C**) Drill tip positioned in the center of the hole; (**D**) Drilling of the hole after aligning the drill tip with the X-ray beam for a “bullseye shot” and resulting drilling trajectory indicated by green circles.

As a pilot application, a module to practice freehand distal locking of intramedullary nails was developed (Figure 1). Users can practice the steps of perfect circle alignment, drill tip centering and drilling on an artificial tibia bone model (tibia right distal, 3108, SYNBONE AG, Zizers, Switzerland). The system features a miniaturized model of an intraoperative image intensifier (C-arm) (1) with an artificial X-ray imaging engine generating radiation-free simulated X-rays. Based on the alignment of the C-arm and by pressing a footswitch, reconstructed radiographs are generated out of a computed tomography (CT) volume (Figure 2). The C-arm’s position and orientation are calculated by the tracking system using the optical camera (2) and reference markers (1) with cylindrical holes [22,23]. A virtual representation of an intramedullary tibia nail is superimposed in the radiographic image and displayed on the computer’s screen (3). Nail geometry and alignment can be digitally configured providing the option to generate random nail orientations to challenge the trainee. Moreover, no physical nail needs to be incorporated in the system. Users can practice the task of perfect circle alignment by orienting the C-arm until a circular projection of the targeted hole is achieved in the X-ray image (Figure 2A,B). The module is complemented by an angulated power drill (4) mimicking the use of a radiolucent drill drive. Position and orientation of the drill is monitored via another attached optical reference markers (4). A virtual drill bit is visualized in the X-ray image to exercise drill tip positioning and drill alignment (Figure 2C). The subsequent drilling is tracked, and the resulting drilling trajectory is displayed in the X-ray image (Figure 2D).

The system accuracy was assessed using a high precision optical motion capture system (Aramis SRX, GOM GmbH, Braunschweig, Germany). The test yielded an accuracy and precision of 1.41 ± 0.75 mm (mean ± standard deviation) for drill tip position and 0.14 ± 0.17° for drill orientation.

**Figure 3:**
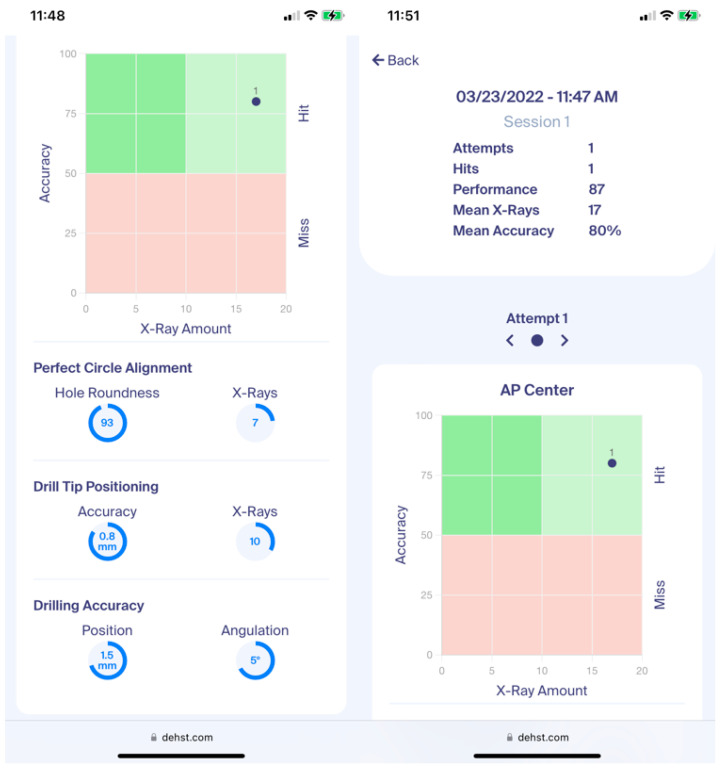
DEHST web application for data collection, analytics and training assessment.

Several performance metrics are generated for training assessment. The result of perfect circle alignment is specified by the hole roundness (100% = perfectly circular hole projection; 0% = no projection visible) and the number of taken radiographs. Drill tip centering is assessed by the achieved accuracy (distance of the drill tip to the center of the hole in mm) and the number of taken radiographs. Drilling is evaluated based on the offset of the drill trajectory from the nail hole center (in mm) and the angular deviation from the hole axis (in °). The ratio of hit and miss attempts (hit/miss ratio) is evaluated considering a hit and thus successful distal interlocking as achieved when the angular deviation is less than 10° and the positioning offset is within the range of 2 mm. These values are based on the nail hole geometry and may be adapted for other nails. Additional assessment parameters are calculated, namely the total number of X-ray images, overall accuracy (combination of angular deviation and positioning offset) and a performance score (ratio of accuracy and number of X-rays). All results are uploaded to an associated web application (Figure 3) and personalized via an individual QR code with a mobile phone. Users can access training data and progress through the web application.

### 2.2. Participants and Study Groups

Fifty-three participants at the AO Davos Courses from 28 November to 3 December 2021 volunteered for this study and were assigned into novices (*n* = 29, orthopaedic trauma residents without surgical experience in distal nail interlocking) and experts (*n* = 24, consultants or chief surgeons with surgical experience of more than 10 nail osteosynthesis treatments). All participants signed an informed consent agreeing to use their blinded information for research purposes. Institutional review board approval was not necessary as neither patient charts were used nor human subjects were involved.

### 2.3. Protocol and Construct Validity

All participants were recruited at the exhibition booth during the AO Davos Courses 2021. After an approximately one-minute system introduction, the surgical steps for freehand distal nail interlocking according to Medoff [24], later modified by Kelley et al. [25], were explained to all participants in a standardized manner: (1) achieving a perfect circle projection of the central anteroposterior nail hole (Figure 2B) by aligning the C-arm under repeated X-ray imaging; (2) drill tip positioning in the center of the hole under repeated X-ray imaging (Figure 2C); (3) aligning the drill to the X-ray beam for a “bullseye shot” and drilling of the hole (Figure 2D). The exact outcome metrics were not explained to the participants. Prior to each trial, a random virtual nail position was generated out of a set of five different nail position options. The results were fed back to the user via the web application, and a score board presented the participants’ performance in relation to the tested peer group (Figure 1 right).

Training data of all users were collected and processed for construct validity. The following metrics were statistically analyzed between the novice and expert groups: number of X-rays to achieve perfect circle alignment and position the drill tip in the center of the hole, roundness of the nail hole, drill tip position accuracy, angular deviation and positioning offset of drilling, and hit/miss ratio.

### 2.4. Questionnaire and Face Validity

All participants completed a questionnaire containing meta-information, age, gender, handedness, function at their institution, specialization, region, and experience with intramedullary nailing. Furthermore, questions were asked about the realism and training capability of DEHST using a Likert scale consisting of 7 points between “1: absolute not realistic” and “7: perfectly realistic” (Figure 3) [26]. The Likert scale is widely used in validation studies [26,27,28,29]. Moreover, participants were asked eight “agree”, “disagree” or “undecided” questions on the usefulness of DEHST (Table 1). Face validity was evaluated from the results of the questionnaire. This approach is well established and has been used in numerous validation studies of medical training devices [1,4,5,6,7,8,9,10,11,12,13,14,15].

### 2.5. Statistical Analysis

Statistical analysis was performed using SPSS software package (v.27, IBM SPSS, Armonk, NY, USA). Shapiro-Wilk test was applied to screen normality of the data distribution. Mann-Whitney-U, Chi-Square and Fischer’s Exact tests were applied to detect significant differences between the groups. Level of significance was set to 0.05 for all statistical tests.

## 3. Results

### 3.1. Participants

The novice group consisted of 29 participants (all residents; 16 females and 13 males) with a mean age of 28.2 (range, 26–32) years. Novices were based in: Asia-Pacific: 1; North America: 1 and Europe: 27. Four were left-handed. The expert group consisted of 24 participants (7 chiefs, 6 senior consultants, 11 consultants; 2 females and 22 males) with a mean age of 35.3 (range, 29–48) years. Experts were based in: Latin America: 3; Middle East: 2; North America: 2; Asia Pacific: 1 and Europe: 16. Two were left-handed.

### 3.2. Face Validity

**Figure 4:**
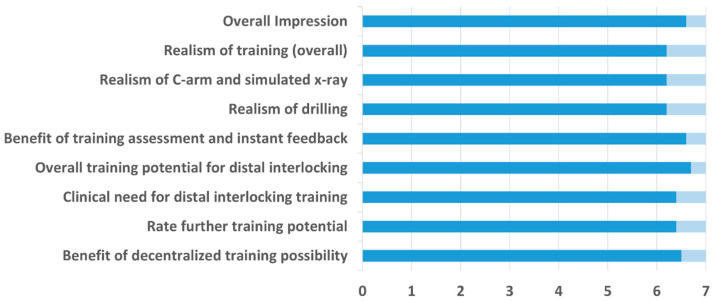
Results from the questionnaire of all participants. 1: “absolute not realistic”; 7 “perfectly realistic”.

The results of the Likert scale in the questionnaire are depicted in Figure 4. The first four questions aimed to evaluate the realism of the training device which was rated 6.3 on average (range 4–7). The other questions aimed to assess training potential and need for distal interlocking training, which was rated 6.5 on average (range 5–7). In general, novices and experts rated all questions between 6 and 7 on average with no significant differences between the groups for any question, *p* ≥ 0.236. Only realism of drilling and clinical need for distal interlocking training were once rated below 4 (3) by two novices separately. The eight agree/disagree/undecided statements are depicted in Table 1. Answers were not significantly different between the groups, *p* ≥ 0.113. The opinion of the participants regarding questions 1, 2, 4, 7 and 8 was consistent (agree). The remaining questions, however, were answered heterogeneously. Moreover, 34% of the participants agreed that there are certain disadvantages of the simulated training and specified this in the open answer section of the questionnaire. Statements of the expert group included: bone too soft, drill machine too light, maybe costs, foot pedal needs too long to react, fixed leg, and C-arm handling needs improvement. Statements of the novices included: power drill different from reality, no continuous fluoroscopy, size of C-arm too small, and device does not simulate a real operation.

### 3.3. Construct Validity

**Figure 5:**
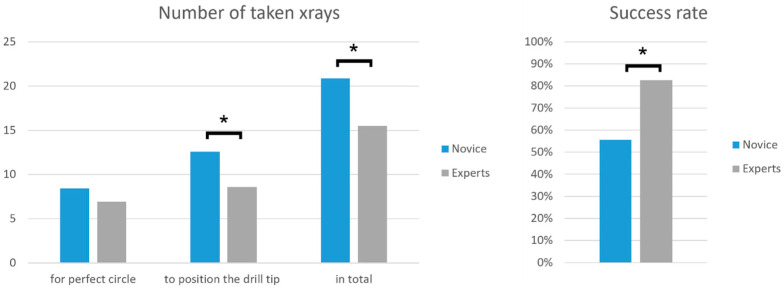
Comparison of novice (blue) and expert (grey) performance based on the captured metrics. Numbers of taken X-rays (**left**) and success rate (hit/miss ratio) (**right**). Stars indicate significant differences between the groups.

Construct validity was evaluated by comparing the obtained metrics of the two groups (Figure 5). Hole roundness of the perfect circle was 88.3 ± 6.5% for novices and 91.0 ± 3.9% for experts, *p* = 0.208. Novices and experts needed a similar number of X-rays (8.4 ± 3.7 versus 6.9 ± 3.0, *p* = 0.171). Accuracy of the positioned drill tip in the center of the perfect circle was similar between novices and experts (1.3 ± 0.7 mm versus 1.3 ± 0.9 mm, *p* = 0.792). However, the novices needed significantly more X-rays to position the drill tip in the center of the hole (12.6 ± 4.8 versus 8.6 ± 3.9, *p* = 0.005). Consequently, total number of X-rays to complete the surgical task was significantly higher for the novices compared to the experts (20.9 ± 6.4 versus 15.5 ± 5.3, *p* = 0.003). Final drill tip position accuracy in relation to the center of the virtual nail hole was similar between the groups (novices: 1.4 ± 3.6 mm versus experts: 1.1 ± 0.6 mm, *p* = 0.254). Angulation of the drill in the virtual nail hole was similar between the groups (novices: 7.7 ± 3.6° versus experts: 6.1 ± 3.1°, *p* = 0.099). The hit/miss ratio was significantly higher in the expert group (novices hit: *n* = 15; 55.6% versus experts hit: *n* = 19; 83%, *p* = 0.040).

## 4. Discussion

The current study evaluated construct and face validity of the new DEHST system for distal nail interlocking. The findings confirm its construct validity—being the first step of the validation cascade—as significant and plausible differences were found between experts and novices. Furthermore, face validity was established as the study participants considered the presented exercise as a realistic representation of distal nail interlocking and stated that the tested device is a useful tool that they could recommend to colleagues. Furthermore, they expressed high perceived realism and considerable training potential for distal interlocking and other surgical training procedures using fluoroscopic guidance.

A key finding of the study is the difference between the groups regarding number of used X-rays. Although novices and experts needed the same number of X-rays to achieve the perfect circle, experts needed significantly less X-rays to position the tip of the drill in the center of the hole. However, roundness of the perfect circle and accuracy of the positioned drill tip were not different between the groups. The significantly higher number of novices missing the drill hole is another important indicator for the validity of the training device. If a surgeon misses the window for safe passing of the drill bit through the hole, the drill will either be completely deflected from its trajectory or will be forced into the nail hole creating an oval-shaped channel, which reduces the primary stability of the nail construct, especially in poor bone quality. By training young surgeons with the DEHST device such incorrect drill channels may be avoided. However, future studies are needed to evaluate this in cohorts of untrained and trained novices. The results of the current study revealed some distinct weak points for novices that can be considered in further medical training. Moreover, they underline the importance of the self-assessment aspect of DEHST allowing instant feedback and pointing out individual weak aspects during training (e.g., drill tip position and drilling). In other future DEHST applications this feature may help identify training needs in young surgeons.

We regarded a surgeon with over 10 interventions as an “expert”, which is a lower number compared to other studies where participants needed over 50 surgeries to be considered as experts [27,28,30]. This number is debatable as not only surgical expertise but also three-dimensional understanding and manual dexterity play a role in performing this surgery task. The number was chosen as a surgeon performing more complex procedures—such as hip arthroplasty—is considered as “professional” after 50 operations [31] and interlocking of an intramedullary nail is only one surgical step of a complex procedure. Furthermore, it is challenging to set a threshold score when using a Likert scale to establish face validity, however, the average points between 6 and 7 for all answers in the current study were considered as highly acceptable. This is in line with other studies evaluating face validity and considering 5 out of 7 points [26,32] or 7 out of 10 points [33] as acceptable to establish face validity.

DEHST is constructed in modular fashion to seamlessly integrate other surgical trainings steps requiring X-rays guidance. All participants indicated high future training potential (6.4 on the Likert scale) in the questionnaire. Mainly experts had suggestions for possible further training applications involving the acetabulum, pelvis, scaphoid, ankle, and spine. There are certainly some downsides of the evaluated training device commented by 38% of the participants in the open section of the questionnaire. For example, the drill power tool was mentioned as being too light and different from a surgical power drill, however, this seems to be acceptable as the used power tool simulates appropriately the drilling procedure and reduces significantly the costs of the system. Furthermore, the bone was rated as too soft—this will be considered in future DEHST versions. An interesting statement by a novice addressed the fact that continuous fluoroscopy was not possible. Although the device is theoretically able to perform live fluoroscopy, we chose to deactivate this feature and follow the method described by Medoff [24] and Kelley et al. [25]. Live fluoroscopy produces large amounts of radiation that should be avoided and is out of the DEHST training scope. Another novice stated that the size of the C-arm is too small. However, the system is constructed as portable on-side training device accepting the imperfect replication of a real operation room scenario. A larger C-arm would significantly reduce the mobility of the device. A further novice stated that the training system does not simulate a real operation. This comment relates to the fact that no soft tissues were present in the simulated tibia. However, the specimen may be covered with a fabricated foam simulating a real human ankle and foot. Using a soft tissue cover makes the surgical task more demanding and could hypothetically lead to a larger discrepancy between experts and novices. Due to test setup reasons during the AO Davos Courses 2021 with only limited time of the participants, we waived the use of a foam cover. In future efficacy studies of DEHST this feature will be included.

### Methodological Considerations

This study has some limitations to be considered when interpreting the results. First, the number of participants was comparably low. However, 53 participants from all continents except Africa, with 24 experts in the field of trauma surgery giving lectures at the AO Davos Courses represent a robust basis for the current study. This participant number is similar to several other studies evaluating medical training devices [24,28,29,30,31,32,33,34,35,36,37,38,39,40,41,42]. Second, some novices showed up in groups at the exhibition booth, thus a learning effect by observing other participants could not be excluded. In real-life young surgeons sometimes perform the surgical step without prior teaching of the skill. Therefore, performance of the novices might have been overestimated in the current study. However, we decided to standardize the introduction to the training device and the surgical task for both groups. Third, due to the test situation at the AO Davos Courses 2021 with several people at the exhibition booth, participants might have behaved differently as they would in a controlled laboratory situation. For this reason, we decided to exclude the time needed to perform each task as a performance metric from the study. Further studies under controlled laboratory conditions are in planning to address these limitations.

## 5. Conclusions

The evaluated Digitally Enhanced Hands-On Surgery Training system for training of distal nail interlocking yielded high scores in terms of training capability and realism. Furthermore, construct validity was established as it reliably discriminates between experts and novices. All participants see a high overall training potential of the concept, as the system may be easily adapted to various other surgical core tasks of daily clinical practice.

## Figures and Tables

**Table 1:** Face validity: agree/disagree/undecided statements regarding the training simulator. Values are presented in *n* (%).

Question	Agree	Disagree	Undecided
1. The training simulator is useful for procedural training of distal nail interlocking	53 (100)	0 (0)	0 (0)
2. The training simulator (distal interlocking) should be offered to all novices for pre-training before performing surgery on real patients	47 (89)	0 (0)	6 (11)
3. The training simulator (distal interlocking) should be obligatory for pre-training novices before performing surgery on real patients	32 (60)	9 (23)	12 (17)
4. The training simulator (distal interlocking) should be recommended for any orthopaedic trauma resident to improve his/her skills individually	50 (94)	0 (0)	3 (6)
5. The training simulator (distal interlocking) should be integrated into the current curriculum of the specialization program of orthopaedic surgeons (e.g., FMH, Facharzt)	28 (53)	11 (21)	14 (26)
6. There are certain disadvantages in the simulator training method. Please specify:	18 (34)	14 (26)	21 (40)
7. I would like to have the simulator in my institution	51 (96)	0 (0)	2 (4)
8. I would recommend the simulator to my colleagues	52 (98)	0 (0)	1 (2)

## Data Availability

All data relevant to the study are included in the article.
